# ALK-Positive Anaplastic Large Cell Lymphoma: A Diagnostic Dilemma for the Otolaryngologist in a Resource Poor Setting

**DOI:** 10.1155/2021/3756742

**Published:** 2021-11-01

**Authors:** Nicholas Figaro, Rickhi Ramoutar, Rodolfo Arozarena, Dawn Meyers, Solaiman Juman

**Affiliations:** ^1^Department of Clinical Surgical Sciences, University of the West Indies, Eric Williams Medical Sciences Complex, Champs Fleur, Trinidad and Tobago; ^2^Department of Pathology, San Fernando General and Teaching Hospital, San Fernando, Trinidad and Tobago

## Abstract

Anaplastic large cell lymphoma is a rare subtype of non-Hodgkin's lymphoma. The morphological diversity with which its anaplastic cells confer make the diagnosis of this hematological entity extremely challenging to the pathologist in a resource poor setting. We present a case of a 35-year-old male with a nasopharyngeal mass and cervical lymphadenopathy and the adversities faced by out otolaryngology department with obtaining the diagnosis of ALK-positive anaplastic large cell lymphoma.

## 1. Introduction

Anaplastic large cell lymphoma (ALCL) was first described by Stein et al. in 1985 as a peripheral T cell lymphoma [1, 2]. Previously unrecognized, this lymphoma is uniquely characterized by the cohesive proliferation of large pleomorphic lymphoid cells expressing CD 30 [3, 4]. ALCL accounts for <5% of all non-Hodgkin's lymphoma, and despite being primarily a nodal disease, it can involve extranodal sites such as the skin, stomach, small intestine, and head and neck regions [5]. The otolaryngologist requires a high index of suspicion and clinical awareness to promptly diagnose and treat rare pathologies such as ALCL. This case report showcases an anaplastic lymphoma kinase- (ALK-) positive anaplastic large cell lymphoma presenting with persistent cervical lymphadenopathy and a nasopharyngeal mass.

## 2. Case Description

A 35-year-old man of Afro-Trinidadian descent was referred to the Eric Williams Medical Science Complex after experiencing 2 months of unresolving, mildly tender neck swellings, and progressive bilateral nasal obstruction. The patient stated that he was employed as a sanitary worker and believed he had contracted a job-related infection that would have eventually and uneventfully passed. Despite the patient's initial conservative approach, he grew concerned of the neck lumps and nasal obstruction and as such sought medical attention from a general practitioner. He was prescribed a two-week course of broad spectrum antibiotics that was deemed ineffective. Approximately, two weeks later, he presented to our emergency department with worsening neck swelling and malaise.

The patient had no comorbidities, no recent travelling history, nor was he in contact with any animals. However, he admitted to a 15-pack year history of cigarette smoking.

**Figure 1:**
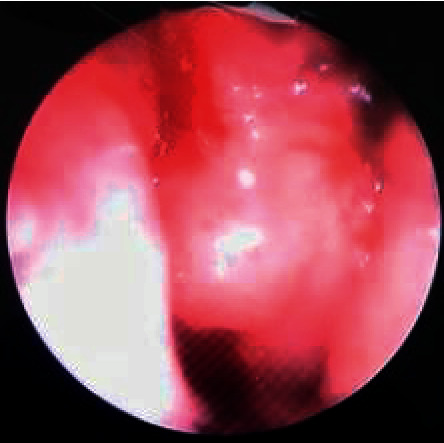
Flexible nasopharyngoscopy showing the polypoid mass in the nasopharynx.

Physical examination on palpation revealed right-sided, mildly tender lymphadenopathy in levels 2–4, and the largest of which was 2 cm in greatest dimension. Oral cavity, ear, and cranial nerve examinations were all unremarkable. Flexible nasopharyngoscopy demonstrated an enlarged, polypoidal mass arising from the nasopharyngeal wall (Figure 1). Paraclinical findings for the human immunodeficiency virus, hepatitis B, monospot, Mantoux, and human T-lymphotropic virus were all negative. Serum electrolytes, renal function, and blood glucose were all within limits. The white blood cell count and hemoglobin were 7.0 × 10^9^/L and 13.5 g/dL, respectively, both within normal limits. However, the serum lactic acid dehydrogenase level was moderately elevated at 465 U/L.

**Figure 2:**
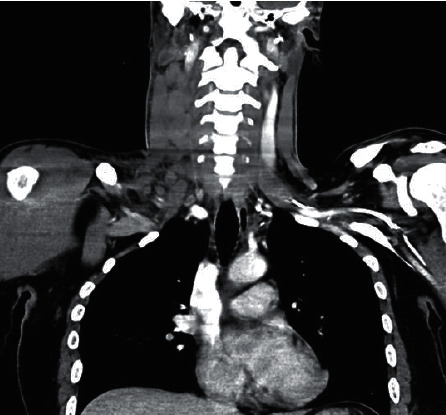
Coronal CT imaging of the neck showing right-sided cervical lymphadenopathy.

**Figure 3:**
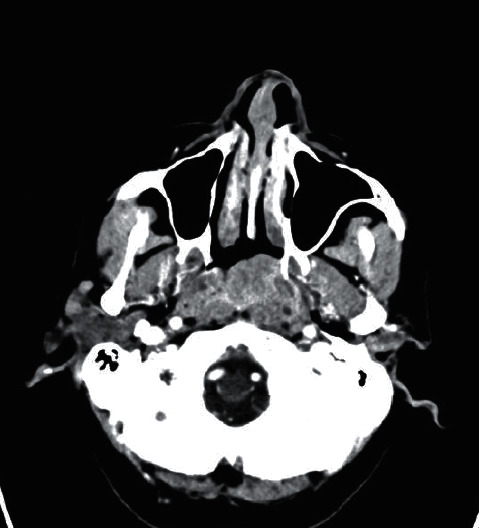
Axial CT imaging of the head showing the nasopharyngeal mass.

A contrast-enhanced computed tomography (CT) of the patient's neck confirmed right-sided cervical lymphadenopathy of the lateral compartment (Figure 2) and a nasopharyngeal mass (Figure 3). Fine needle aspiration cytology of the cervical lymph nodes was inconclusive. Subsequently, a biopsy of the nasopharyngeal mass as well as an excisional lymph node biopsy were performed in the operating room. Histological analysis, namely, hematoxylin and eosin staining revealed reactive hyperplasia of both the cervical lymph node and postnasal mass. The patient was later discharged to the outpatient clinic with a two-week course of azithromycin, a tapered dose of oral prednisolone and topical corticosteroid spray.

**Figure 4:**
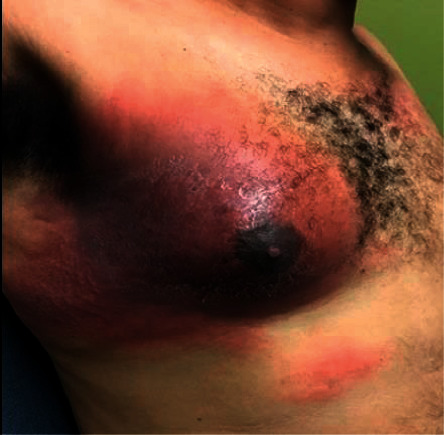
Erythematous skin lesion.

**Figure 5:**
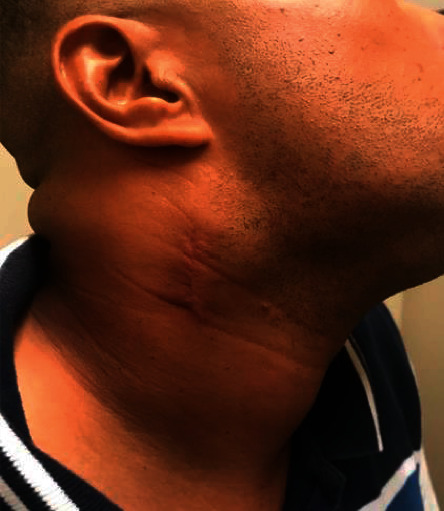
Progressive neck swelling.

**Figure 6:**
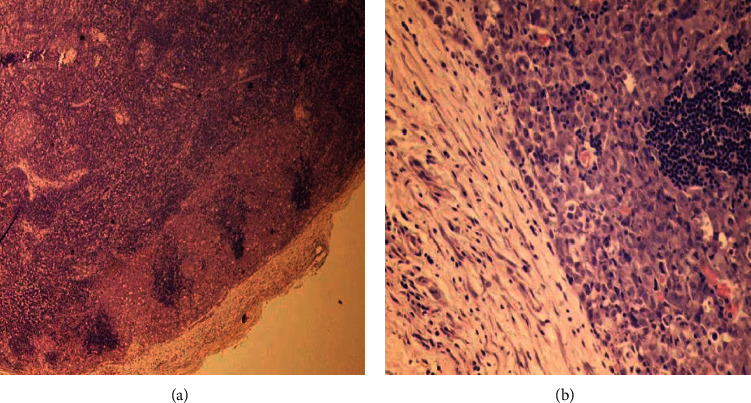
(a) At low magnification, focal effacement of lymph node architecture by cohesive infiltrate of neoplastic cells, extending from the subcapsular sinuses into the paracortical region. (b) At high magnification, large pleomorphic neoplastic cells with abundant eosinophilic cytoplasm, pleomorphic nuclei, and frequent mitotic figures, including a “hallmark” cell with horseshoe-shaped nuclei (blue arrow).

**Figure 7:**
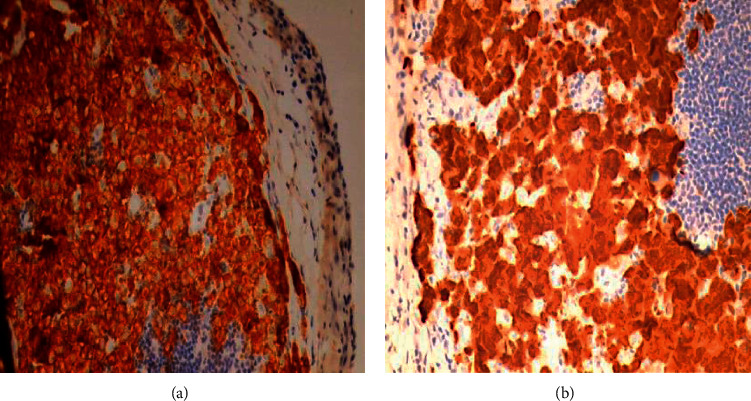
(a) At high magnification, strong, uniform CD 30 positivity in the cytoplasmic membranes and Golgi regions of the neoplastic cells. (b) At high magnification, diffuse cytoplasmic and nuclear staining for ALK.

At his outpatient clinic follow up visit, the patient reported fevers, night sweats, an erythematous skin lesion distributed over his right chest (Figure 4), and an increase in the size of the right-sided cervical lymph nodes (Figure 5). Despite the prior benign histological result, a high degree of suspicion of malignancy arose. The histological blocks were obtained from our institution's pathology lab and subsequently submitted to another public tertiary health facility for immunohistochemical analysis to be performed. The pathological analysis yielded focal effacement of the lymph node architecture by large cells with abundant eosinophilic cytoplasm, pleomorphic nuclei, and frequent mitotic figures (Figures 6(a) and 6(b)). The immunohistochemical test results demonstrated strong and diffuse, positivity for CD30 and ALK in the tumour cells (Figures 7(a) and 7(b)). The tumour cells were negative for CD5, CD4, CD8, PAX 5, and CD68. As such, the features supported a diagnosis of an ALK-positive anaplastic large cell lymphoma with a null cell phenotype.

**Figure 8:**
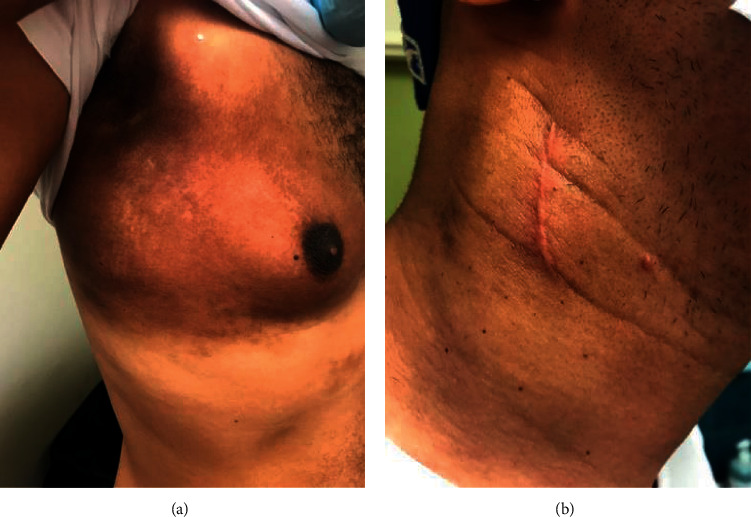
(a) Resolution of skin lesion. (b) Resolution of right cervical lymphadenopathy.

Based on the diagnosis of lymphoma, the patient was referred to the oncology service. The oncologist was able to urgently stage the patient with further CT imaging and bone marrow aspirate evaluation. The 35-year-old male was staged as Ann Arbor stage 3B ALK-positive ALCL. His International Prognostic Index was high to immediate risk with 43% and 5-year overall survival with 55% complete response. A chemotherapy regime of cyclophosphamide, doxorubicin, vincristine, and prednisone began the subsequent week. The chemotherapy course was well received by the patient as the cervical lymphadenopathy, nasal mass, skin lesions, and constitutional symptoms resolved (Figures 8(a) and 8(b)). The patient is currently alive and has been in satisfactory condition 24 months since initial diagnosis.

## 3. Discussion

The differential diagnosis of adult cervical lymphadenopathy is extensive; however, when coupled with a nasopharyngeal lesion, it narrows the focus to several key diagnoses. Approximately 90% of patients with this combination will be diagnosed with nasopharyngeal carcinoma. The remaining 10% can be more commonly divided among lymphomas, adenoid cystic carcinoma, adenocarcinoma, mucoepidermoid carcinoma, neuroendocrine carcinoma, plasmacytoma, melanoma, and secondary metastatic carcinomas [6, 7]. The diagnosis of ALK-positive ALCL presenting in an adult with a nasopharyngeal mass and cervical lymphadenopathy is rare. Based on our review of the literature, this is the third reported case, and the two previously described cases occurred in young women of Asian descent [1, 8].

The World Health Organization classifies ALCL into two distinct clinical entities, a systemic form and a cutaneous form, with the former being further subdivided by ALK protein expression or absence [5, 9, 10]. A translocation between chromosome 2 and chromosome 5 [*t* (2; 5) (*p*23; *q*35)] is one of the distinctive features that occur in 40–60% of ALCL patients [2, 4, 10]. The translocation triggers the integration between nucleophosmin (NPM) and the transmembrane receptor tyrosine gene, ALK. The described synergism eventually leads to the expression and upregulation of a unique chimeric NPM-ALK protein [3, 5, 11]. The exhibition of the ALK protein is an important diagnostic and prognostic feature of ALCL [4, 10, 12].

Lymphoma presenting as a postnasal space lesion is somewhat unusual. Approximately 1 in 10 patients diagnosed with non-Hodgkin's lymphoma will have extranodal spread at the time of presentation, namely, in the skin, lung, bone, soft tissue, and gastrointestinal tract [13]. Within this subset, approximately 20% of these patients will have involvement of Waldeyer's ring similar to this case, where the pharyngeal tonsil of this patient was affected [13]. In retrospect, the red brown maculopapular rashes on the patient's chest are characteristic cutaneous manifestations of primary systemic ALCL and are commonly seen in approximately 20% of patients with extranodal spread [14].

This case report serves as a reminder that adult adenoidal tissue is not benign when accompanied by sinister features [15]. Clinical signs and symptoms that are causes for concern include cervical lymphadenopathy, otalgia, facial pain, epistaxis, and otitis media with effusion [13, 15]. Additionally, radiological imaging depicting asymmetrical nasopharyngeal tissue growth is generally not associated with innocuous disease, as per the index case [16, 17]. Notwithstanding the initial report given by the pathologist, a high degree of suspicion should have been maintained in the pursuit of an alternative diagnosis given the initial presenting complaint.

The diagnostic dilemma that ALCL poses occurs as a direct consequence of the morphological diversity that anaplastic cells confer. As a result, it is not uncommon for ALCL to be misdiagnosed as melanoma, metastatic carcinoma, sarcoma, and several other hematopoietic malignancies [4, 12]. Sinusoidal disease is a well-known trademark of ALCL, yet it can be subtle and supplemented with prominent reactive morphological features like follicular hyperplasia [4]. These illusive histological features can explain the results of reactive hyperplasia obtained in the index case by our institution's pathologist on H&E staining.

Immunohistochemistry, flow cytometry, and fluorescence in situ hybridization have become invaluable tools in the diagnosis, classification, and management of lymphomas [18]. Unfortunately, they are only available at few public tertiary health institutions in Trinidad and Tobago. As such, clinical awareness of the disease, thorough clinical examination, cooperation, and efficient communication between the clinician and pathologist are paramount to establish an accurate and timely diagnosis. In our setting, there is a policy for obtaining a second opinion from another institution's pathologist; however, the onus is on the principal clinician to initiate the request in pursuit of a precise diagnosis.

Moving forward, we propose that an interinstitutional multidisciplinary meeting should be performed with similar challenging cases. This format not only provides an avenue for patients to access a greater number of experts but it improves service coordination and mitigates the time to final diagnosis and management implementation.

## 4. Conclusion

The unavailability of appropriate investigative techniques can make an uncommon clinical entity like ALK-positive ALCL a diagnostic nightmare. However, maintaining a high degree of clinical suspicion is essential in establishing such rare diagnoses. Moreover, the relationship between clinician and pathologist is crucial to facilitate a prompt and accurate diagnosis for this subset of patients.

## Data Availability

No data were used to support this study.
